# Development and Validation of the EspaiJove.net Mental Health Literacy (EMHL) Test for Spanish Adolescents

**DOI:** 10.3390/ijerph17010072

**Published:** 2019-12-20

**Authors:** Pere Castellvi, Rocío Casañas, Victoria-Mailen Arfuch, Juan José Gil Moreno, María Torres Torres, Carlos García-Forero, Dyanne Ruiz-Castañeda, Jordi Alonso, Lluís Lalucat-Jo

**Affiliations:** 1 School of Medicine, Universitat Internacional de Catalunya, 08195 Sant Cugat del Vallés, Spain; pere.castellvi.obiols@gmail.com (P.C.); cgarciaf@uic.es (C.G.-F.); 2 Research Department, Centre Higiene Mental Les Corts, Grup CHM Salut Mental, 08029 Barcelona, Spain; victoriaarfuch@gmail.com (V.-M.A.); lluis.lalucat@chmcorts.com (L.L.-J.); 3 Child and Juvenile Mental Health Centre of Les Corts and Sarrià- Sant Gervasi, Centre Higiene Mental Les Corts, Grup CHM Salut Mental, 08029 Barcelona, Spain; juanjo.gil@chmcorts.com (J.J.G.M.); maria.torres@chmcorts.com (M.T.T.); 4 Fundación para la Investigación Biosanitaria en Andalucía Oriental (FIBAO), 04009 Almería, Spain; dyanneruiz@hotmail.com; 5 Health Services Research Unit, Institut Hospital del Mar d’Investigacions Mèdiques (IMIM), 08003 Barcelona, Spain; jalonso@imim.es; 6 CIBER en Epidemiología y Salud Pública (CIBERESP), 28029 Madrid, Spain; 7 Department of Experimental and Health Sciences (DCEXS), Pompeu Fabra University (UPF), 08003 Barcelona, Spain

**Keywords:** measurement, mental health literacy, adolescence

## Abstract

There is evidence of the effectiveness of implementing mental health literacy (MHL) programs. However, there are substantial limitations in the instruments available for measuring MHL. This study aimed to develop and validate the EspaiJove.net MHL test (EMHL) for Spanish adolescents by assessing its psychometric properties. The development of the EMHL test was conducted using item pool generation and a pilot study. A convenience sample of students aged 13–15 years (*n* = 355) participated in the validity study. Reliability was assessed for internal consistency and via test-retest. Convergent validity was evaluated by comparing the effect sizes among known groups with different levels of mental health knowledge, the correlation with mental health-related instruments, and the item discrimination index. A final version of a 35-item EMHL test was obtained with two parts: (i) a binary choice format (yes/no) for the identification of mental disorders; (ii) a multiple choice question with four possible answer options. Internal consistency was acceptable in the first part (Cronbach’s alpha = 0.744; Guttman’s lambda 2 = 0.773) and almost acceptable in the second part (Cronbach’s alpha = 0.615; Guttman’s lambda 2 = 0.643). The test-retest evaluation supported the stability of the test (first part, ICC = 0.578; second part, ICC = 0.422). No ceiling and floor effects were found. The EMHL test scores discriminated between known groups with different levels of mental health knowledge and it is associated with several-related constructs of MHL. Conclusions: The EMHL test is a relevant measure for assessing MHL in adolescents into Spanish context with acceptable validity and stability.

## 1. Introduction

It is estimated that 75% of all people suffering from a mental disorder have experienced the onset at 25 years old [1,2] and 50% during adolescence [3]. Promoting mental health to prevent mental disorders and their consequences is one of the main goals in public health [4]. A lack in mental health literacy (MHL) is associated with mental illness and delays in seeking help, so an increase in the community’s MHL is needed to empower the community in improving mental health [5,6].

MHL is defined as “a set of knowledge and beliefs about mental disorders which aid their recognition, management or prevention” [5]. MHL involves: (a) the ability to recognize the development of mental disorders, (b) knowledge and beliefs about risk factors, the causes of mental disorders and how to prevent them, as well as (c) knowledge of how to seek professional help and effective available treatments [5].

Some MHL interventions for adolescents and young people have been developed in recent years in several countries [7,8,9,10,11]. These interventions suggest an improvement in mental health knowledge, in facilitating monitoring and help-seeking, an increase in the self-recognition of mental disorders, and an improvement and reduction in mental health-related stigma. In Spain, the MHL program “EspaiJove.net: a space for mental health” (EspaiJove.net) [12] has been developed in Barcelona in secondary schools.

A systematic review of the measurement properties of tools measuring MHL [13] showed that there are only two specific instruments targeting adolescents: one was only for depression [14] and the other one focused on improving beliefs and attitudes towards mental health [15]. This highlights the need for the development, evaluation, and validation of tools addressing mental health knowledge specifically for adolescents who are vulnerable to developing a mental illness. So, there is a validation gap in measuring MHL categories and the related psychometric properties of these instruments. Furthermore, no validated MHL measures addressing knowledge of positive/good mental health have been developed [16]. Developing an assessment in a test format that comprehensively evaluates the main contents of the MHL (negative and positive mental health, and help-seeking) would allow a more specific and rigorous assessment of MHL levels in adolescents, and the effectiveness of MHL interventions.

To our knowledge, no specific MHL questionnaire has been properly validated. The aim of this study is to develop and validate the EspaiJove.net mental health literacy (EMHL) test for the assessment of MHL in Spanish. The EspaiJove.net intervention consists of a universal MHL intervention which aims to promote mental health, prevent mental disorders, and facilitate help-seeking behaviors among secondary school students in the Spanish context.

## 2. Materials and Methods

The EMHL test is a maximum performance test (criterion-referenced test—CRT) based on thematic content from EspaiJove.net. The item pool generation from EspaiJove.net thematic module contents include: (1) The concepts of mental health and mental disorders, (2) mental health multidisciplinary team network and the use of health services, (3) healthy and risk behaviors in mental health, (4) social skills and antisocial behavior, bullying and cyber-bullying, (5) anxiety, (6) depression, (7) self-harm and suicidal behaviors, (8) eating disorders, (9) alcohol and substance use, and (10) psychotic disorders.

**Figure 1:**
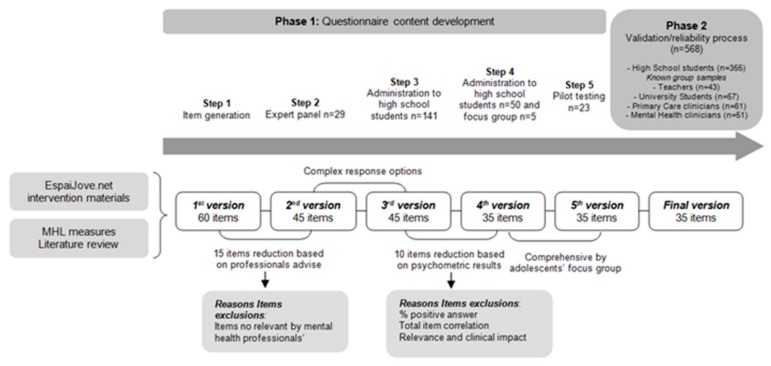
Description of the phases of development of the EspaiJove.net Mental Health Literacy (EMHL) test.

The EMHL test development process involved two phases, as shown in Figure 1. The content development of the EMHL test was developed using a literature review and focus groups. For more information, see Appendix A.

### 2.1. The EMHL Test Score

To obtain the EMHL total test score for each part of the test, the formula (A-E)/(n-1) is used, where A = number of correct answers, E = number of errors (including missing values), and n = number of options for each item. Then, for the first part of the EMHL test, the formula is (A-E)/(2-1), and for the second part it is (A-E)/(4-1), where each correct answer adds one point to the total score, and each incorrect answer results in zero points (Uncorrected total score) [17]. To facilitate the interpretation of results, both sections were converted into continuous scales ranging from 0 to 10 (corrected scores) using this formula (U-m)/(M-m), where U = Uncorrected total score, m = the minimum score allowed, M = the maximum score allowed. So, for the first part of the EMHL test, the formula is ((U − (−15))/(15 − (−15))) × 10 which is ((U + 15)/30) × 10, and for the second part it is ((U − (−6.67))/(6.67 − (−6.67))) × 10, which is ((U + 6.67)/13.34) × 10. A higher score means greater mental health knowledge.

### 2.2. Validation of the Psychometric Properties of the EMHL Test

#### 2.2.1. Sample

The validation process was performed through the administration and analysis of the final version of the EMHL test to a non-randomized convenience sample of high school students aged 14/15y(*N* = 355) in 6 schools in Barcelona, Spain, and the informed consent signed by both adolescents and parents. Exclusion criteria included: (1) Students with special educational needs and/or with cognitive problems; and (2) no understanding of Spanish or Catalan. Nurses and psychologists who were members of the EspaiJove.net team informed the participants about the content of the study and administered the EMHL test.

Written informed consent from all adults participating (teachers, university students, primary care physicians and nurses and mental health professionals), high school students, and the parents of all students participating in the study was requested.

#### 2.2.2. Main Validity Measures

We hypothesized specific variables to associate them with the level of MHL, with varying degrees of strength.

Stigma was measured using two questionnaires: (1) the Scaling Community Attitudes toward the Mentally Ill (CAMI) Spanish version [18] which consists of 40 items divided into four dimensions (authoritarianism; benevolence; community mental health ideology and social restrictiveness). We only used the authoritarianism dimension (10 items). For the social restrictiveness dimension, the 4 questions on the future of the RIBS were chosen, since both work on the same concepts. Higher scores mean greater agreement in engaging in the stated attitude; (2) the reported and intended behavior scale (RIBS) consists of 8 items, the first four of which are designed to assess the prevalence (past and current) of behaviors in each of the four contexts (1. living with; 2. working with; 3. living nearby; and 4. being in a relationship with someone with a mental health problem) while items 5–8 ask about intended (future) behaviors within the same contexts [19]. We selected four items from 5 to 8 (future behaviors). Higher scores indicate greater agreement with engaging in the stated behaviors. We hypothesized that higher mental health knowledge would attract less stigma.

Mental health. The strengths and difficulties scale (SDQ) was used [20]. The SDQ consists of 25 items which generate scores along five dimensions: emotional symptoms, conduct problems, hyperactivity/inattention, peer problems, and prosocial behavior (positive mental health). We hypothesized that adolescents with higher emotional symptoms and peer and conduct problems would have lower mental health knowledge, and more prosocial behaviors would have higher mental health knowledge. No a priori relationship will be found for hyperactivity/inattention because there is no item in the EMHL test regarding this construct.

Health-related quality of life (HRQoL). The 5-level EQ-5D is a brief, multi-attribute, generic, preference-based health status measure [21,22]. We used the Spanish version of EQ-5D-5L and time trade-off preference values from the Catalan general population [23]. EQ-5D-5L scores range from negative values to 1, with higher scores indicating better health status, and 0 being equal to death. We hypothesized that more anxiety / depression as indicated by EQ-5D dimension 5 would mean lower mental health knowledge; however, the remaining HRQoL dimensions of the EQ-5D will not show this relationship.

Bullying and cyberbullying. We developed a 4-item scale to assess bully victims and the bullying behaviors of perpetrators specifically for this study. Two items assess whether an adolescent has been bullied or cyberbullied and two items have bullying or cyberbullying behaviors. We hypothesized that adolescents who have been bullied or bullies themselves would have lower mental health knowledge.

#### 2.2.3. Known-Groups Validity Assessment

We recruited high school teachers, nursing and psychology university students, primary care physicians and nurses, and mental health professionals (psychiatrists, psychologists and nurses) (*n* = 213). We hypothesized that some groups, in particular health professionals and teachers, would have significantly higher mental health knowledge than high school students.

#### 2.2.4. Statistical Analysis

##### Reliability

Missing values were assessed. The distribution of the item responses from complete responders was analyzed in order to detect highly skewed distributions and the floor or ceiling effects of correct answers. The internal consistency index for CRTs was calculated using the phi (lambda) coefficient [24,25] as an estimate of consistency. This coefficient is specific to CRTs and is interpreted as a Cronbach’s alpha coefficient and 95% confidence intervals (95% CI) [26], obtaining values between 0 and 1. One month test-retest reliability was assessed with the intraclass correlation coefficient (ICC) two-way random model and 95% CI that tested for absolute agreement between the first and second administration of the scale. Values below 0.4 were considered as poor, between 0.40 and 0.59 as fair, between 0.60 and 0.74 as good, and over 0.75 as excellent [27]. In the case of negative ICC values, each item will be deleted, and the Alpha coefficient will be assessed again to measure the consistency of the EMHL test.

##### Convergent Validity

The ability of the EMHL test’s uncorrected total score was assessed to distinguish among different groups. Differences across known groups were assessed with the ANOVA parametric test. The magnitude of the association was estimated with the effect size (ES) to compare average differences in the MHL mean between subgroups in categorical variables. The cutoffs and the interpretation of ES were low (|0.20| ≤ ES ≥ |0.50|), moderate (|0.50| < ES ≥ |0.80|), and high (ES > |0.80|) [28,29]. In the case of continuous measures, the magnitude of the association was assessed by cut-offs for Pearson correlation coefficients: very weak (< 0.20), weak (≥ 0.20–< 0.40), moderate (≥ 0.40– <0.60), strong (≥ 0.60– < 0.80), and very strong (≥ 0.80) [30]. Significance tests were all evaluated at the 0.05 level. Additionally, we assessed Alpha coefficient for all validity measures administered in our sample to observe if there is an error measurement in the proxies used to assess MHL construct.

The item discrimination index was also assessed, and it evaluates how well an individual question sorts the sample out between those who have mastered the material and those students who have not. It is based on comparing the performance of the extreme groups (low and high) in the test scores. The number of participants who have been successful in the high group (mastered) was compared to those in the low proficiency group (non-mastered). We selected 36% of the sample. The discrimination capacity of each item was assessed by these cut-offs: items that must be deleted (*D* ≤ 0.0), inadequate (*D* > 0.0– < 0.20), low (*D* ≥ 0.20– < 0.30), acceptable (*D* ≥ 0.30– < 0.40), and strong (*D* ≥ 0.40) discrimination.

Statistical analyses were conducted using the Statistical Package for the Social Sciences (SPSS, Inc., Chicago, Ill., USA) version 22.0 [31] and Microsoft Office Excel 2007 (Microsoft Corporation, Redmond, Washington, DC, USA).

## 3. Results

Among the 355 high school students, 178 (50.1%) were women (four subjects were missing), and 56 (15.8%) were non-Spanish nationals, with mean (SD) age at 14.5 (0.66).

### 3.1. Reliability

**Figure 2:**
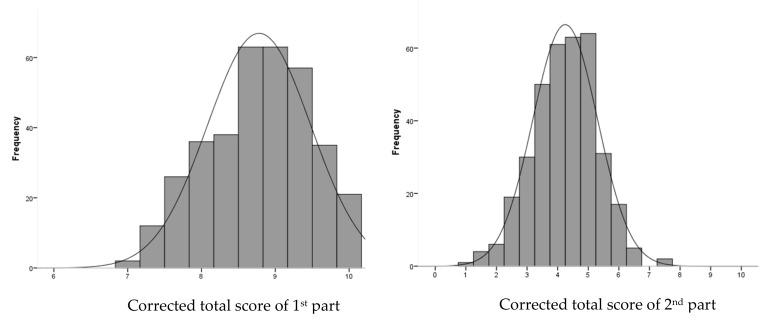
The EMHL test uncorrected and corrected score distribution in high school students sample.

The no-item category was missing. A visual inspection of item response frequencies showed very few skewed distributions in both parts of the EMHL test. Nevertheless, the Kolmogorov-Smirnov normality test showed as significant for uncorrected and corrected total scores (*p* < 0.001) in both total scores, which was most likely due to the large sample size studied. We assumed that both parts of the EMHL test have a normal distribution of total scores (see Figure 2). Median (25–75 percentile) and mean (SD) corrected total scores for the first part were 8.7(8.3–9.3) and 8.8(0.7), and for the second part were 4.5(3.5–5) and 4.3(1.1), respectively. The first part of the items revealed varying levels of difficulty with a large range of correct responses (28.9% to 97.7%), and the second part items were more demanding, ranging from 11.9% to 83.0% of correct responses. Thus, only 5.9% of the total sample scored the maximum possible (ceiling effect) score for the first part and 0.6% for the second part of EMHL test. For the ceiling effect, the proportion of the total sample that scored the minimum possible score was both 0.3% for the first and second part of the EMHL test. The first part of the EMHL test showed internal consistency values above ≥0.70, Cronbach’s alpha was 0.744 and Guttman’s lambda 2 was 0.773. However, the second part was below < 0.70, 0.615 of Cronbach’s alpha and 0.643 of Guttman’s lambda 2, respectively. Corrected item-to-total correlations in each item ranged from 0.162 to 0.526 for the first part and ranged from −0.206 to 0.411 for the second part (Table 1). The items with negative values in ICC (items 2 and 4) were deleted separately and Cronbach’s alpha was assessed again. Results showed an increase in Alpha coefficient (Cronbach’s α when deleting item 2 = 0.660; Cronbach’s α when deleting item 4 = 0.644). However, the increase has not been considerable. Score test-retest reliability using uncorrected scores and measured with the ICC were fair for both parts of the EMHL test: first part, ICC (95% CI) = 0.578(0.326–0.736), *p* < 0.001, and second part, ICC (95% CI) = 0.422(0.072–0.639), *p* = 0.012.

### 3.2. Convergent Validity

Table 2 shows the EMHL uncorrected total test scores by known groups. Results show significant differences across subgroups (F (4563) = 140.459; *p* < 0.001; *η^2^* = 0.499). For the first part of the EMHL test, all groups had a significant and moderate magnitude of association compared with high school students (range ES, 0.466 to 0.640; *p* < 0.001). In the second part of the EMHL test, teachers, university students and primary care physicians and nurses had a significant and moderate magnitude of association compared with high school students (range ES, 0.485 to 0.692; *p* < 0.001), and a significant and high magnitude of association when we compared with mental health professionals (ES, 0.812; *p* < 0.001) (see Table 2).

Table 3 shows correlations between several scales and EMHL uncorrected test scores. In the first part of the EMHL test, high school students with higher uncorrected scores for MHL had a weak strength and significant correlation of CAMI’s authoritarianism dimension (*r* = −0.246; *p* < 0.01), and a very weak but significant correlation for HRQoL in the anxiety/depression domain of EQ-5D-5L (*r* = −0.122; *p* < 0.05), and in the emotional symptoms domain of SDQ (*r* = 0.140; *p* = 0.009).

In the second part of the EMHL test, high school students with higher uncorrected scores for MHL had a weak strength and significant correlation for CAMI’s Authoritarianism dimension (*r* = −0.222; *p* < 0.01) and RIBS’ future behavior (*r* = <0.201; *p* < 0.01), and a very weak and significant correlation in the conduct problems domain of SDQ (*r* = −0.121; *p* < 0.05), HRQoL in the self-care domain of EQ-5D-5L and bullying behaviors (*r* = −0.130; *p* < 0.05). Other domains and scales were not significantly related to both parts of the total scores from the EMHL test.

Finally, we assessed if there is an error measurement using Alpha coefficients in each validity measures. Results suggest there has been error measurement in most of them (CAMI’s Authoritarianism dimension: 0,141; SDQ total difficulties score: 0.469; EQ-5D: 0.596) (see Table 3).

All items of the first part and almost all items of the second part of the EMLH test show a powerful discrimination index using the 36% of the sample and comparing the subsample with the highest performance levels and the lowest (mastered vs. non-mastered). In the second part of the test, only item 18 (delusions) has an acceptable level of discrimination (*D* = 0.33), and item 4 (healthy behaviors) has low levels of discrimination (*D* = 0.24) (see Table 4).

## 4. Discussion

This study described the development of the EMHL test and its psychometric properties in a sample of Spanish adolescents, showing good validity properties and stability over time. The EMHL test is a measure created to assess the effectiveness of EspaiJove.net intervention, and more generally, to evaluate the knowledge and belief about mental health and mental disorders, knowledge of the risk behaviors of a mental disorder and the help-seeking behaviours. Accordingly with the developmental process, the EMHL test has been developed to deliver a clinically relevant measurement adapted to the Spanish context, supported by extensive recommendations by mental health experts and taking into account the opinion of adolescents, using non-offensive and adolescent-adapted vocabulary which was delivered via the content on the EspaiJove.net universal MHL intervention. Regarding the validity of the results, the EMHL test demonstrated it was capable of distinguishing between known groups with different levels of mental health knowledge, correlated with some variables related to mental health, related-behaviors and HRQoL, and it has the capacity to differentiate between those adolescents who have mastered the materials of the EspaiJove.net and those who have not. So, these results suggest that this test will be appropriate for the assessment of its efficacy after the intervention. However, some reliability measures showed slightly low scores in the EMHL test.

### 4.1. Reliability of the EMHL Test

The score distribution for the EMHL test showed that it has an appropriate ceiling and floor effect and stability over time, where less 6% of the adolescents answered all the items correctly in both parts of the EMHL test, respectively. Regarding reliability, these results suggest that this test can be used over time for the assessment of the efficacy of interventions aimed at increasing MHL among Spanish adolescents, such as the EspaiJove.net intervention, and among the adult population with an estimated higher level of MHL. However, internal consistency showed low results for the second part of the EMHL test, although it almost reached the ≥0.70 value which is considered to be acceptable. Internal consistency ranges from poor to fair in both parts of the EMHL test, where the same results have been demonstrated in corrected item-to-total correlations. Furthermore, two items (item 2—where to go for help; item 4—healthy behaviours) have negative values recommending to delete both items. However, the opinions of mental health professionals considering both items as clinically relevant, recommend to keep them in the EMHL test. According to a previous systematic review of the quality of developed MHL instruments [13], out of fifteen MHL instruments developed that assessed internal consistency or reliability, five of them demonstrated poor quality properties, two in the adolescent population for assessing mental disorders and depression, respectively [14,15], and six for the general population [32,33,34,35,36,37], one for schizophrenia, two for depression, and three for mental disorders, respectively. One hypothesis regarding these low values is that the EMHL test broadly covers a wide range of mental health constructs. In fact, this instrument asks about mental health services, healthy and risky behaviors, conduct problems and antisocial behaviors among peers, and several mental disorders and problems such as anxiety, mood, eating and behavioral disorders, substance use and psychotic disorders, and self-harm and suicidal behaviors. So, it is most likely that the capacity of the EMHL test to cover the main clinically relevant questions to promote mental health and to prevent mental disorders may have considerably decreased its internal consistency. Nevertheless, during the development process, skilled mental health professionals and high school students were asked about the items that they considered being more relevant to be used with adolescents for improving mental health. Other hypothesis is that the second part of the EMHL test uses multiple choices including distracting items and based on stereotypes, prejudices and erroneous statements about mental health increasing the difficulty of the test and the variability of their answers and decreasing its reliability. So, in conclusion, although some reliability values showed poor properties, we consider that the opinion of skilled professionals and targeted adolescent population prevails over statistical analyses for the final inclusion of the items in the EMHL test.

### 4.2. Convergent Validity of the EMHL Test

Both parts discriminated between wide ranges of groups (adolescents, postgraduate students, high school teachers, primary care professionals and mental health professionals) with different levels of mental health knowledge. The discriminative capacity of the EMHL test between these groups makes it possible to conduct studies among youths, even in the adult population and to conduct studies that assess the effectiveness of MHL interventions aimed at increasing the level of mental health knowledge up to that of mental health professionals which would be the gold standard of MHL and expertise. Although most validity measures showed poor or fair reliability, such as stigma-related with CAMI’s authoritarianism dimension, mental health symptoms and HRQoL, the EMHL test also demonstrated significant correlations between many mental health-related constructs, as our a priori hypotheses regarding the pattern of correlations between MHL and mental health-related stigma, emotional symptoms, anxiety and depression HRQoL, conduct problems and bullying behaviors were generally satisfied. As predicted, adolescents with higher MHL levels had less mental health-related stigma, anxiety and depression HRQoL, conduct problems and bullying behaviors, and higher emotional symptoms. Unexpectedly, higher self-care HRQoL correlated with higher MHL levels. This unexpected correlation is probably related to the positive mental health items in the EMHL test. These results suggest that higher scores of MHL using the EMHL test have an impact on these constructs.

### 4.3. Strengths and Limitations

Our study has several strengths, including a large sample size, as well as the fact that the EMHL test addresses a gap in the assessment of adolescent MHL. Methodologically, this study proposes an evaluation method for MHL that has been scarcely explored so far. The CRTs are generally used in education to find out to what extent each subject dominates the criterion of interest. In this case, the selection of items is not based on individual differences and the variability of answers but on the purpose of the test: to identify those students who manage the domain and those who do not. Therefore, the CRTs’ decisions are based on acceptable performance rather than the relative position [38].

On the other hand, our study has some limitations. Although this instrument has been developed for the Spanish context, the EMHL test has been developed with adolescents living in Barcelona, so further validation should be assessed in other cities, regions and settings. Secondly, the broad scope of the EMHL test and the complexity of multiple choice questions could diminish its reliability. However, we consider that adolescents who know key questions about mental health prevention, and which they consider to be useful, are more important and clinically relevant than the consistency of the test assessed statistically.

## 5. Conclusions

As a result, this study provides a new valid instrument for the evaluation of MHL interventions. Although the EMHL test is only used for the EspaiJove.net intervention, it could be a promising tool to inspire other MHL measurements.

## Figures and Tables

**Table 1:** Percentage of floor and ceiling effects and item-total score correlations.

Item	Floor *N(%)*	Ceiling *N(%)*	Item-Total Score Correlation
**First part of the EMHL test**			
1 (Schizophrenia)	67(19.0)	286(81.0)	0.309
2 (Diabetes)	12 (3.4)	341 (96.6)	0.337
3 (Phobia)	60 (17.0)	293 (83.0)	0.272
4 (Asthma)	13 (3.7)	340 (96.3)	0.405
5 (Bipolar disorder)	24 (6.8)	329 (93.2)	0.340
6 (Bulimia)	127 (36.0)	226 (64.0)	0.411
7 (Cerebral palsy)	172 (48.7)	181 (51.3)	0.526
8 (Bronchitis)	4 (1.1)	349 (98.9)	0.394
9 (Alzheimer disease)	251 (71.1)	102 (28.9)	0.287
10 (Depression)	38 (10.8)	315 (89.2)	0.374
11 (Chickenpox)	8 (2.3)	345 (97.7)	0.408
12 (Down’s Syndrome)	199 (56.4)	154 (43.6)	0.503
13 (Epilepsy)	153 (43.3)	200 (56.7)	0.354
14 (Anorexia nervosa)	79 (22.4)	274 (77.6)	0.437
15 (Substance dependence)	85 (24.1)	268 (75.9)	0.162
**Total First Part**	2 (0.6)	21 (5.9)	* 0.744
			+0.773
**Second part of the EMHL test**			
1 (Definition of mental health)	282 (79.9)	71 (20.1)	0.132
2 (Where to go for help)	87 (24.6)	266 (75.4)	−0.206
3 (Who develops a mental disorder)	72 (20.4)	281 (79.6)	0.249
4 (Healthy Behaviors)	257 (72.8)	96 (27.2)	−0.133
5 (Night rest)	102 (28.9)	251 (71.1)	0.268
6 (Cannabis / alcohol consumption)	308 (87.3)	45 (12.7)	0.401
7 (Bullying)	139 (39.4)	214 (60.3)	0.127
8 (Cyberbullying)	110 (31.2)	242 (68.6)	0.229
9 (Social skills)	191 (54.1)	162 (45.9)	0.236
10 (Suicidal ideation friend / family)	151 (42.8)	202 (57.2)	0.144
11 (Self-injury)	109 (30.9)	244 (69.1)	0.267
12 (Suicide Behavior Alert)	60 (17.0)	293 (83.0)	0.178
13 (Depression)	270(76.5)	83 (23.5)	0.407
14 (Eating Disorders)	294 (83.3)	59 (16.7)	0.256
15 (Start of Eating Disorders)	270 (76.5)	83 (23.5)	0.238
16 (Characteristics of Eating Disorders)	281 (79.6)	72 (20.4)	0.411
17 (Symptoms Schizophrenia)	269 (76.2)	84 (23.8)	0.301
18 (Delusions)	261 (73.9)	92 (26.1)	0.039
19 (Psychotic episode)	311 (88.1)	42 (11.9)	0.396
20 (Alcohol dependence)	243 (68.8)	110 (31.2)	0.376
**Total Second Part**	1 (0.3)	2 (0.6)	* 0.615
			+0.643

EMHL = EspaiJove.net Mental Health Literacy test; * Cronbach’s alpha; +Guttman’s Lambda 2.

**Table 2:** EspaiJove.net mental health literacy (EMHL) test scores according to “known groups”, using uncorrected total scores.

Subgroups	N	Mean (SD)	Effect Size (ES)	*p*
**First part of the EMHL test**				
High school students (ref.)	355	7.07 (4.96)	---	<0.001
Teachers	43	11.56 (3.42)	0.466	<0.001
University students	57	12.44 (2.32)	0.570	<0.001
Primary care physicians and nurses	61	13.56 (1.90)	0.654	<0.001
Mental health professionals	51	13.55 (2.37)	0.640	<0.001
**Second part of the EMHL test**				
High school students (ref.)	355	−1.06 (1.48)		<0.001
Teachers	43	0.60 (1.52)	0.485	<0.001
University students	57	1.32 (1.34)	0.645	<0.001
Primary care physicians and nurses	61	1.86 (1.56)	0.692	<0.001
Mental health professionals	52	3.13 (1.53)	0.812	<0.001

EMHL test = EspaiJove.net Mental Health Literacy test; ES = effect size; ref = reference group; SD = standard deviation.

**Table 3:** Correlations between the EMHL test scale using uncorrected total score and other construct-related scales.

Validity Measures			First Part of the EMHL Test		Second Part of the EMHL Test	
	N	Alpha Coefficient	Pearson Correlation	*p*-Value (2-Tailed)	Pearson Correlation	*p*-Value (2-Tailed)
Stigma-related (CAMI)	344	0.141	−0.246**	<0.001	−0.222**	<0.001
Stigma-related (RIBS)	352	0.794	−0.094	0.079	−0.201**	<0.001
Emotional Symptoms (SDQ)	352		0.140**	0.009	0.032	0.553
Conduct Problems (SDQ)	353		−0.095	0.074	−0.121*	0.023
Hyperactivity/inattention (SDQ)	353		0.044	0.406	−0.028	0.596
Peer problems (SDQ)	353		−0.041	0.442	0.006	0.909
SDQ total difficulties score	352	0.469	0.025	0.645	−0.030	0.576
Prosocial (SDQ)	353	0.611	0.029	0.588	−0.023	0.664
Bully victim	352		0.016	0.764	0.068	0.201
Bully perpetrator	351		−0.050	0.348	−0.130*	0.014
Mobility (EQ-5D-5L)	353		−0.045	0.401	0.000	0.995
Self-care (EQ-5D-5L)	353		0.075	0.160	0.130*	0.014
Usual activities (EQ-5D-5L)	353		−0.057	0.286	0.093	0.081
Pain/discomfort(EQ-5D-5L)	353		−0.021	0.696	−0.051	0.341
Anxiety/depression (EQ-5D-5L)	353		−0.122*	0.021	−0.062	0.246
EQ-5D-5L total score	353	0.596	0.018	0.741	0.070	0.193
EQ-5D-VAS	352		−0.083	0.120	−0.059	0.273

CAMI= Community Attitudes toward Mental Illness; EMHL = EspaiJove.net Mental Health Literacy test; EQ-5D-5L= 5-level EQ-5D version; EQ-5D-VAS= EQ-5D Visual Analogue Scale; RIBS= Reported and Intended Behaviour Scale; SDQ= Strength and Difficulties Questionnaire. **. Correlation is significant at the 0.01 level (2-tailed). *. Correlation is significant at the 0.05 level (2-tailed).

**Table 4:** Item discrimination index of the EMLH test comparing the highest performance levels (mastered) and the lowest (non-mastered) subsamples and using 36% of the total sample.

**First Part of the EMHL Test**	***D***
1 (Schizophrenia)	0.96
2 (Diabetes)	0.85
3 (Phobia)	0.92
4 (Asthma)	0.88
5 (Bipolar disorder)	0.88
6 (Bulimia)	1.12
7 (Cerebral palsy)	1.25
8 (Bronchitis)	0.83
9 (Alzheimer disease)	0.78
10 (Depression)	0.93
11 (Chickenpox)	0.86
12 (Down’s Syndrome)	1.21
13 (Epilepsy)	1.02
14 (Anorexia nervosa)	1.07
15 (Substance dependence)	0.83
**Second Part of the EMHL Test**	***D***
1 (Definition of mental health)	0.52
2 (Where to go for help)	0.53
3 (Who develops a mental disorder)	1.03
4 (Healthy Behaviors)	0.24
5 (Night rest)	1.05
6 (Cannabis / alcohol consumption)	0.76
7 (Bullying)	0.87
8 (Cyberbullying)	0.99
9 (Social skills)	0.90
10 (Suicidal ideation friend / family)	0.84
11 (Self-injury)	1.06
12 (Suicide Behavior Alert)	1.01
13 (Depression)	0.87
14 (Eating Disorders)	0.50
15 (Start of Eating Disorders)	0.54
16 (Characteristics of Eating Disorders)	0.71
17 (Symptoms Schizophrenia)	0.66
18 (Delusions)	0.33
19 (Psychotic episode)	0.55
20 (Alcohol dependence)	0.95

EMHL = EspaiJove.net Mental Health Literacy test; *D* = Item Discrimination Index.

## Appendix A. Phase 1: Questionnaire Content Development of the EMHL Test

The development of the EMHL test was based on: (1) a literature review of MHL measures for adolescents; and (2) content analysis of the discourse that emerged from 6 focus groups held by trained child and adolescent mental health professionals (psychiatrists, psychologists and nurses) and high school students aged 14/15 years.

The questionnaire content development phase included 5 steps:

Step 1: Item pool generation. The first version of the EMHL test was developed from the thematic content of the EspaiJove.net program resulting in 60 items (version 1) (part 1: 15 items; part 2: 45 items). The part 1 consisted of a binary choice format (yes/no) for the recognition of mental disorders from a list of 15 different diseases (15 items), and part 2 has multiple choice questions with four possible answer options, in which only one is correct. Incorrect answers were considered as distracting items and were based on stereotypes, prejudices and erroneous statements about mental health. The distracting items were selected from focus groups held with adolescents (*n* = 39) and then developed by EspaiJove.net researchers and reviewed by mental health professionals.
Step 2: We then conducted 6 focus groups with a total of 29 mental health professionals (expert panel) from four public child and juvenile mental health centers to explore: (i) clinical relevance; (ii) mistakes in wording (questions and answers for each item); and (iii) comprehensiveness and offensiveness. Semi-structured cognitive interviews were implemented to guide the discussions and were recorded. As a result, an initial selection of 45 items (version 2) (part 1: 15 items; part 2: 30 items) from the preliminary version 1 was developed.
Step 3: The 45-item EMHL test version 2 (part 1: 15 items; part 2: 30 items) was used in five high school classrooms (*n* = 141) in three different public schools. The objective was to examine (i) item relevance by adolescents, (ii) its comprehensiveness and level of difficulty, (iii) its offensiveness, and (iv) the feasibility of the test (version 3).
Step 4: Finally, the EMHL test was used with 3rd grade high school students (*n* = 50) and a focus group (*n* = 5) with the purpose of improving comprehensiveness and vocabulary. The last 35-item test (version 4) (part 1: 15 items; part 2: 20 items) was selected for the pilot study.
Step 5: Pilot testing study. The final 35-item EMHL test version (part 1: 15 items; part 2: 20 items) was piloted among 3rd grade high school students (*n* = 23) to assess its comprehensiveness and vocabulary. A validation process was performed on this EMHL test version (version 5).

From the analyses of the results we finally selected 35 items (version 3) (part 1: 15 items; part 2: 20 items) deleting each question that presented two out of the three criteria as follows: (i) Psychometric properties (<0.20 in total item score correlations), (ii) Knowledge level (>50% of positive answers); (iii) Relevance (less relevant items as considered by mental health professionals).
